# Integrated pan-cancer analysis of ADM’s role in prognosis, immune modulation and resistance

**DOI:** 10.3389/fimmu.2025.1573250

**Published:** 2025-06-03

**Authors:** Yunhuan Liu, Caicun Zhou

**Affiliations:** ^1^ Department of Oncology, Shanghai Pulmonary Hospital, School of Medicine, Tongji University, Shanghai, China; ^2^ Department of Oncology, Shanghai East Hospital, School of Medicine, Tongji University, Shanghai, China

**Keywords:** adrenomedullin (ADM), pan-cancer analysis, lung adenocarcinoma (LUAD), immune modulation, resistance

## Abstract

**Introduction:**

Adrenomedullin (ADM), a multifunctional peptide, has been implicated in various inflammatory and autoimmune diseases. However, its role in cancer, particularly in NSCLC, remained under-explored. This called for a pan-cancer analysis of ADM, investigating its expression, genomic alterations, prognostic value, immune associations, and relations with drug sensitivity to provide insights into its potential as a therapeutic target and biomarker.

**Methods:**

ADM expression data from normal and tumor tissues was retrieved and analyzed through HPA and Timer 2.0 online platforms. Genetic alterations, copy number variations (CNVs), and methylation patterns were analyzed using cBioPortal and GSCA platforms. The data for survival analysis was extracted from TCGA and GEO database and analyzed through GEPIA and PrognoScan online platforms. ADM’s correlations with immune checkpoint genes, immune cell infiltration, MSI, and TMB were evaluated using data from Timer and TCGA via R. Drug sensitivity analysis was performed with GDSC and CTRP databases, supported by network visualizations. IHC staining was conducted on LUAD patients’ samples to assess ADM’s relationship with EGFR-TKI resistance and immune microenvironment.

**Results:**

ADM was widely expressed across normal tissues, with high levels in adipose tissue, endocrine organs, digestive and reproductive systems. Pan-cancer analysis revealed that ADM expression was upregulated in multiple cancer types, including CESC, ESCA, GBM, HNSC, KICH, KIRC, LUSC, PCPG, THCA, and UCEC, and correlated with advanced pathological stages in THCA, KIRP, and HNSC. Furthermore, high ADM expression was significantly linked to poor prognosis in patients with LGG, LUAD, MESO, THYM, LIHC, HNSC, GBM, KICH, KIRP, CESC, PAAD, and STAD, while its negative influence on OS and RFS was validated in LUAD. In addition, ADM exhibited genetic alterations, including amplification and deep deletion across multiple cancer types. Strong and consistent positive correlations were witnessed between ADM and several immune checkpoint genes, including CD274 (PD-L1), CD276, TNFRSF18, TNFSF9, and PVR in pan-cancer analysis, indicating its role in the development of suppressive immune microenvironment and T cell exhaustion. Besides, ADM showed significant correlations with immune cell infiltration, and TMB/MSI, highlighting its role in immune regulation and its potential as a predictive biomarker for immunotherapy. Significantly, ADM expression was correlated with multiple drug sensitivity, particularly chemotherapy and tyrosine kinase inhibitors (TKIs) therapy. Moreover, positive correlations between its expression and EGFR-TKI resistance, CD8^+^ T cell infiltration and tumor proportion score (TPS) in LUAD were validated in patients’ samples, emphasizing its potential in guiding personalized therapy.

**Discussion:**

This pan-cancer analysis revealed ADM’s pivotal role in progression, immune modulation, and therapeutic response, especially in LUAD. ADM held promise as a prognostic biomarker and a potential therapeutic target in immune modulation and resistance management. Future research should focus on experimental validation and elucidation of ADM-mediated pathways, which might provide novel insights into cancer biology and improve clinical outcomes

## 1 Introduction

Lung cancer, primarily non-small cell lung cancer (NSCLC), remains the leading cause of cancer-related deaths globally (1, 2). Despite advancement in therapeutic strategies, the prognosis of NSCLC patients was poor due to late-stage diagnosis and tumor aggressiveness (3, 4). The development of chemotherapy, targeted therapy and immunotherapy hugely improved the therapeutic strategies of NSCLC. Particularly, immune checkpoint inhibitors (ICIs) targeting the PD-1/PD-L1 pathway, had shown impressive efficacy by enhancing immune response in NSCLC and multiple other cancer types. However, all these therapeutic progressions had limitations. For example, only 19–45% of patients presented response to anti-PD-1/PD-L1 therapy, underscoring the importance of identifying effective predictive biomarkers (4–6). Furthermore, overcoming resistance to chemotherapy and targeted therapy remained an urgent clinical challenge (7). These limitations emphasized the need for more dynamic and reliable biomarkers to guide personalized treatment.

Adrenomedullin (ADM) is a peptide consisting of 52 amino acids. It was generated by the cleavage of a precursor hormone (8). It served a variety of functions, including vasodilation, regulation of hormone secretion, and promotion of angiogenesis. In addition, ADM demonstrated antimicrobial activity, effectively killing Escherichia coli (E. coli) and Staphylococcus aureus (S. aureus) at low concentrations (9–11). These functions positioned ADM as a critical molecule in maintaining vascular health (9, 12, 13), immune response (14–16), and other physiological processes. ADM played a significant role in the pathogenesis of various diseases, including multiple types of cancer. In the context of cardiogenic shock, ADM alleviated cardiac burden and preserved the vascular barrier by promoting vasodilation, improving hemodynamics, and regulating endothelial cell function (17, 18). In neurodegenerative diseases, ADM interacted with cytoskeletal proteins in neurons, influencing microtubule dynamics and contributing to the regulation of neuronal function (19). Furthermore, ADM had been identified as a potential therapeutic target in neurodegenerative diseases. Studies had shown that ADM expression was linked to mechanisms such as neuronal dysfunction, immune responses, inflammation, apoptosis, and calcium dysregulation in Alzheimer’s disease and vascular dementia (20–22). In sepsis, ADM significantly impacted multi-organ function and immune tolerance by modulating the local immune environment, particularly influencing the response of immune cells and cytokines (11, 23, 24). Moreover, ADM exerted immune protection by regulating the proliferation and differentiation of T and B cells (25, 26). The IMD knockout (Adm2-/-) mouse model revealed that the absence of ADM resulted in an immunosuppressive phenotype, which increased susceptibility to pathogens. Supplementing ADM peptides significantly enhanced the expression of T/B cell-related genes, reduced mortality, and mitigated the lethal risks of infection (27). Besides, as a growth factor, ADM stimulated cell proliferation and promoted progression towards more invasive phenotypes in several cancer types. ADM also exerted anti-apoptotic effects and indirectly suppressed immune responses through its binding protein, complement factor H. Notably, in hypoxic environments—commonly found around solid tumors—ADM was upregulated via HIF-1-dependent pathways and acted as a potent angiogenic factor, facilitating the formation of new blood vessels (28).

As a multifunctional peptide with critical roles in vascular regulation, immune modulation, and cellular growth among other physiological processes, ADM’s involvement in malignancies had not been comprehensively analyzed. To address this gap, an extensive pan-cancer analysis of ADM was conducted. A range of bioinformatics tools were used to explore its expression, genomic alterations, prognostic significance, correlation with immune markers, immune infiltration patterns and drug sensitivity across diverse types of cancer. In addition, immunohistochemical (IHC) analysis was performed to further investigate the expression of ADM and its relation with PD-L1 and CD8 in lung adenocarcinoma (LUAD). Our findings suggested that ADM was intricately linked to immune responses and drug sensitivity, and might serve as a potential prognostic biomarker for multiple cancers. These results underscored the relevance of ADM in cancer biology and highlighted its potential as a therapeutic target.

## 2 Method

### 2.1 ADM expression analysis

To investigate the expression levels of ADM across various tissues, several online databases are utilized. Gene expression data for ADM in normal human tissues was obtained from the HPA (https://www.proteinatlas.org) database. The RNA-seq data for ADM in normal and tumor tissues were retrieved from the TCGA (https://portal.gdc.cancer.gov/) and GTEx (https://gtexportal.org/home/) (29). To assess the correlation between ADM expression and pathological stages, the “Stage plot” function from the GEPIA database (http://gepia.cancer-pku.cn/) was employed.

### 2.2 Genetic alteration analysis of ADM

To explore the genetic alterations of ADM across various cancer types, the cBioPortal platform (http://www.cbioportal.org), which provides comprehensive cancer genetic data and facilitates the analysis of molecular data from cancer histology and cytology studies, was utilized (30). The analysis was performed using gene alteration data from 10,967 samples representing 10,953 pan-cancer patients, sourced from the UCSC Xena database (https://xena.ucsc.edu/) and the International Cancer Genome Consortium (ICGC) data portal (https://www.icgc-argo.org), specifically from the “TCGA pan-cancer Atlas Studies” dataset. The research focused on assessing the mutation landscape of ADM, which included mutation types, mutation frequency, and copy number alteration (CNA). The “Cancer Types Summary” module in cBioPortal was employed to extract data on the various mutations of ADM across different cancer types. In addition, the relationship between ADM expression level and copy number alteration was also examined.

Somatic mutation datasets were obtained from the publicly available TCGA database through the Genomic Data Commons website (https://portal.gdc.cancer.gov/) to analyze the relationship between ADM expression and somatic mutations in lung adenocarcinoma (LUAD). The analysis included RNA-seq data and somatic mutation data for LUAD patients, and the ADM expression levels were stratified into high and low expression groups based on the median ADM expression value. The Maftools package in R was used in this analysis, which allowed for detailed mutation profiling, including variant calling, annotation, and visualization. The clinical data incorporated in this analysis included gender, pathological stage, and survival outcome. The somatic mutations were compared between the high and low ADM expression groups to investigate whether ADM expression influenced the mutational burden or specific mutational signatures in LUAD.

### 2.3 Methylation and copy number variations

To examine genomic alterations associated with ADM, the GSCA database (http://bioinfo.life.hust.edu.cn/GSCA/) was used to analyze the methylation and CNVs. Their correlations with the mRNA expression of ADM were presented. The “mutation” module of GSCA was used to assess the alterations in the ADM gene across different cancer types. The relationship between ADM and methyltransferase genes, DNMT1, DNMT3A, DNMT3B was also analyzed, using the pan-cancer RNA-seq data from TCGA database (https://portal.gdc.cancer.gov/), and the results were exhibited in a heatmap made by Chiplot website (https://www.chiplot.online/) (31).

### 2.4 Prognostic analysis

RNA sequencing data and clinical information for multiple cancer types were retrieved from TCGA, Univariate Cox regression analysis was performed using the “survival” package in R to evaluate the prognostic value of ADM. The hazard ratio (HR) with its 95% confidence interval (CI) was calculated while visualizing the results with forest plots. The forest plots were generated with the “ggplot2” package in R. The survival curves were visualized using the survival analysis module of the GEPIA website (http://gepia.cancer-pku.cn/).

To further validate the relationship between ADM expression and prognosis in LUAD and NSCLC, gene expression data and survival data were utilized, from the GEO database (https://www.ncbi.nlm.nih.gov/geo/). GSE31210, GSE3141 and GSE8894 were analyzed based on ADM expression levels using online platform PrognoScan(http://dna00.bio.kyutech.ac.jp/PrognoScan/index.html). Kaplan-Meier survival curves were plotted and Cox regressions were performed to compare survival outcomes between high and low ADM expression groups.

### 2.5 Immune infiltration analysis

To investigate the relationship between ADM expression and immune checkpoint-related genes, RNA sequencing data of multiple cancer types from the TCGA database (https://portal.gdc.cancer.gov/) was utilized. Specifically, this study focused on the correlation between ADM expression and 33 immune checkpoint-related genes. The correlation analysis was conducted using the “cor.test” function in R. Additionally, the immune infiltration data for six types of tumor-infiltrating immune cells (B cells, CD4^+^ T cells, CD8^+^ T cells, neutrophils, macrophages, and dendritic cells) were obtained from the Timer database (https://cistrome.shinyapps.io/timer/) to further explore the relationship between ADM expression and tumor immune microenvironment. To visualize the correlation matrixes, heatmaps were generated with the “pheatmap” package in R. The heatmap was annotated with asterisks (*) to indicate significant correlations.

The microsatellite instability-high (MSI-H) status and tumor mutational burden (TMB) are currently recognized as promising predictive biomarkers for immunotherapy efficacy. MSI and TMB-related data for various cancers were obtained from the TCGA database (https://portal.gdc.cancer.gov/). The correlation between ADM expression and MSI/TMB was analyzed in R, and radar plots illustrating the correlation between ADM and MSI/TMB were generated using the fmsb package.

### 2.6 Drug response analysis

To investigate the drug sensitivity associated with ADM across various cancer types, the GSCALite platform (http://bioinfo.life.hust.edu.cn/web/GSCALite/) was utilized (32). This platform integrated mRNA expression, mutation, immune infiltration, methylation data from TCGA datasets(https://portal.gdc.cancer.gov/), along with drug resistance data from the GDSC (https://www.cancerrxgene.org/) and CRTP (http://portals.broadinstitute.org/ctrp/) databases. Drug sensitivity data for both ADM and ADM2 were retrieved from GDSC and CRTP to explore the relationship between gene expression and drug response. The drug sensitivity analysis was visualized using the GSCALite platform, generating bubble plots to display the correlation between ADM expression and drug sensitivity. Furthermore, to explore the network of anti-tumor medications related to ADM, the R packages “scales” and “igraph” were used to construct and visualize a network of ADM-associated therapeutic drugs. This network was designed to identify potential drugs that could be influenced by ADM expression, highlighting its role within anti-tumor therapy, offering valuable insights for potential therapeutic strategies.

### 2.7 Co-expression and gene set enrichment analysis

To identify genes co-expressed with ADM, the LinkedOmics database (http://www.linkedomics.org/login.php) was accessed (33). This online platform integrated multi-omics data from the TCGA database (https://portal.gdc.cancer.gov/). The appropriate dataset, the “LUAD cohort”, was selected, then Pearson correlation test was applied to identify genes that were significantly co-expressed with ADM. For functional enrichment analysis, the Gene Set Enrichment Analysis (GSEA) tool was utilized to explore the biological processes and pathways enriched in ADM-associated genes. The results were visualized using the LinkedOmics online platform.

### 2.8 Samples and clinical data

Clinical samples for immunohistochemistry (IHC) were obtained from 10 LUAD patients who underwent biopsy at Shanghai Pulmonary Hospital, affiliated to Tongji University, from December 2020 to October 2023. Written informed consent was obtained from each patient. Paired tissue samples collected before and after the development of resistance to first-generation EGFR-TKI, were included in this study. The resistance to EGFR-TKI was confirmed through pathological testing for mutations and clinical follow-up. The study (NO K21-313Z) was approved by the ethics and licensing committee of Shanghai Pulmonary Hospital. The patients’ response to targeted therapy, was analyzed for its correlation with ADM expression. All clinical protocols adhered to the ethical guidelines outlined in the Declaration of Helsinki.

### 2.9 Immunohistochemistry analysis

Immunohistochemistry staining was performed on FFPE tissue sections to assess the protein expression of ADM. Briefly, after deparaffinization and antigen retrieval, sections were incubated with anti-ADM antibody (1:100, Proteintech, 10778-1-AP), anti-CD274 antibody (1:300, Sevicebio, GB115736), anti-CD8 antibody (1:1500, Sevicebio, GB12068). The staining was visualized using a diaminobenzidine (DAB) kit, and slides were evaluated under a light microscope under ×400 magnification by two independent pathologists. The positively stained areas in the images were marked with arrows. The intensity of staining and the percentage of positive cells were scored to quantify ADM and CD8 expression. Meanwhile, the tumor proportion score (TPS) was determined by calculating the percentage of tumor cells exhibiting partial or complete cell membrane staining for PD-L1.

### 2.10 Statistical analysis

All statistical analyses were performed using R software (version 4.4.1) and GraphPad Prism 8. Correlation analysis between ADM expression and methyltransferase genes, correlation analysis between ADM expression and immune-related factors, and correlation analysis of ADM expression with CD8^+^ T cell infiltration and TPS, were all performed using Pearson’s correlation test. For survival analysis, univariate Cox regression was applied to assess the prognostic value of ADM. A p value of <0.05 was considered statistically significant (*p<0.05, **p<0.01, ***p<0.001, ****p<0.0001).

## 3 Result

### 3.1 Expression profile of ADM in normal human tissues

**Figure 1:**
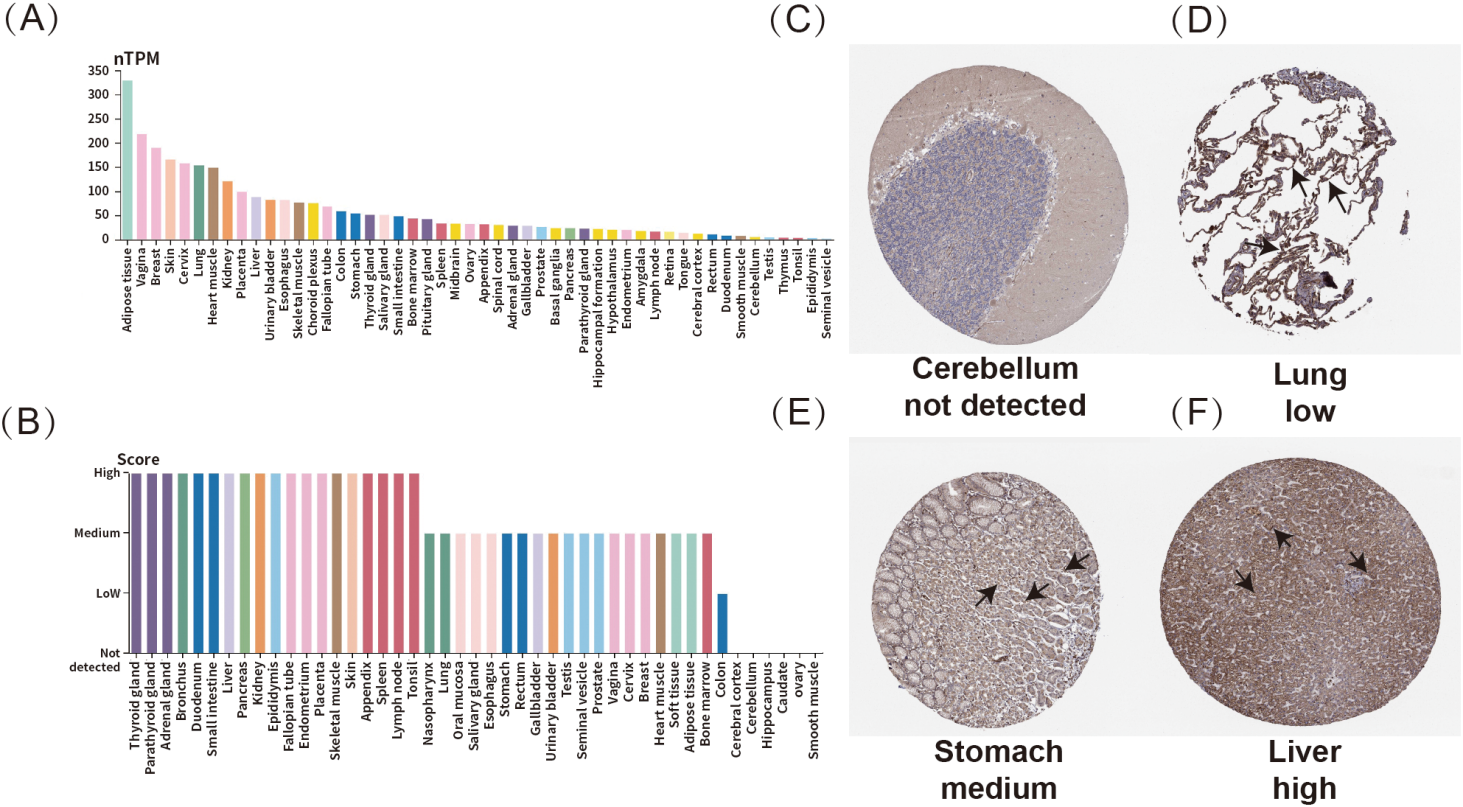
Expression profile of ADM in normal human tissues. **(A)** mRNA expression of ADM across various normal human tissues. **(B)** Protein expression of ADM in normal human tissues. **(C–F)** Representative IHC staining showing ADM expression in normal cerebellum, lung, stomach, and liver. The ADM expression regions were marked with arrows.

To investigate the expression of ADM across various normal human tissues, this research assessed both mRNA and protein expression data using the Human protein atlas (HPA) database. In **Figures 1A-B**, the height of the bars represented the ADM’s RNA or protein expression levels. As shown in **Figure 1A**, ADM mRNA expression was found to be relatively high in adipose tissue, vagina, breast, skin, and cervix. In terms of protein expression, ADM exhibited elevated expression across multiple organ systems, particularly within the endocrine system, including the thyroid; the digestive system, including the duodenum and liver; and the reproductive system, including the fallopian tubes (**Figure 1B**). These findings highlighted ADM’s widespread distribution and its important physiological roles.

Immunohistochemistry analysis revealed varying expression levels of ADM in different tissues. Representative tissue staining results were shown in **Figures 1C–F**, where ADM expression was not detected in the cerebellum (**Figure 1C**). Meanwhile, ADM displayed low expression in the lungs (**Figure 1D**), moderate expression in the stomach (**Figure 1E**), and high expression in the liver (**Figure 1F**). These results further indicated that ADM might play crucial roles in various tissues, with significant variability in expression levels across different organs.

### 3.2 A pan-cancer analysis of ADM expression and tumor characteristics

**Figure 2:**
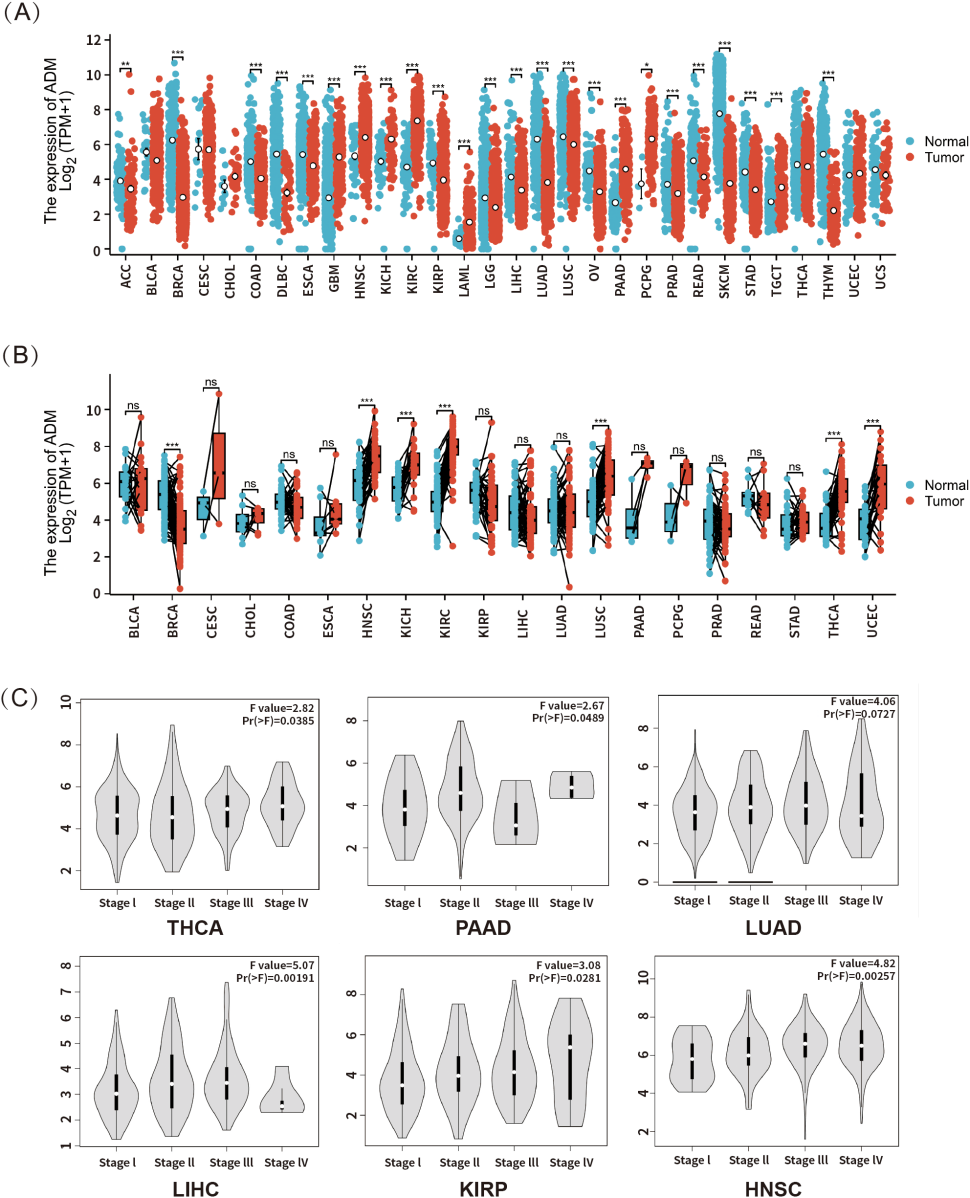
**(A)** ADM mRNA expression in tumor and normal tissues based on data from TCGA and GTEx (n = 17,672). **(B)** ADM expression in tumors and their paired adjacent normal tissues from TCGA (n = 1,394). **(C)** Analysis of the association between ADM expression and cancer pathological stages in THCA, PAAD, LUAD, LIHC, KIRP, and HNSC was performed using the GEPIA database. *p<0.05, **p<0.01, ***p<0.001; ns, not significant.

To investigate the expression of ADM in tumors relative to normal tissues, RNA-seq data from both TCGA and GTEx was integrated. Samples with more than three cases in each group were included in the analysis, while those with smaller sample size were excluded to avoid bias. The analysis revealed significant upregulation of ADM expression in tumor tissues compared to normal ones in certain cancer types, including glioblastoma multiforme (GBM), head and neck squamous cell carcinoma (HNSC), kidney chromophobe (KICH), kidney renal clear cell carcinoma (KIRC/ccRCC), acute myeloid leukemia (LAML), pancreatic adenocarcinoma (PAAD), pheochromocytoma and paraganglioma (PCPG), and testicular germ cell tumors (TGCT). Conversely, ADM expression was significantly downregulated in tumors relative to normal tissues in other cancer types, such as adrenocortical carcinoma (ACC), breast invasive carcinoma (BRCA), colon adenocarcinoma (COAD), diffuse large B-cell lymphoma (DLBC), esophageal carcinoma (ESCA), kidney renal papillary cell carcinoma (KIRP), lower grade glioma (LGG), liver hepatocellular carcinoma (LIHC), lung adenocarcinoma (LUAD), lung squamous cell carcinoma (LUSC), ovarian cancer (OV), prostate adenocarcinoma (PRAD), rectum adenocarcinoma (READ), skin cutaneous melanoma (SKCM), stomach adenocarcinoma (STAD), and thyroid carcinoma (THYM) (**Figure 2A**).

To further validate the differential expression of ADM between tumor and normal tissues, paired RNA-seq data from TCGA for various cancer types were analyzed (**Figure 2B**). In this analysis, significant upregulation of ADM expression was still observed in tumor tissues relative to adjacent normal tissues in head and neck squamous cell carcinoma (HNSC), kidney chromophobe (KICH) and kidney renal clear cell carcinoma (KIRC). Meanwhile, lung squamous cell carcinoma (LUSC), thyroid carcinoma (THCA), and uterine corpus endometrial carcinoma (UCEC) also presented upregulation in ADM expression compared to adjacent normal tissues. However, significant downregulation was only observed in breast invasive carcinoma (BRCA).

Additionally, using the GEPIA database, this study explored the association between ADM expression and cancer stage. A significant correlation was found between ADM mRNA expression and clinicopathological stages in thyroid carcinoma (THCA), pancreatic adenocarcinoma (PAAD), lung adenocarcinoma (LUAD), liver hepatocellular carcinoma (LIHC), kidney renal papillary cell carcinoma (KIRP), and head and neck squamous cell carcinoma (HNSC) (p<0.05) (**Figure 2C**). Overall, ADM mRNA expression generally increased with higher pathological stages. However, in pancreatic adenocarcinoma (PAAD), ADM expression showed considerable fluctuation across stages, while in lung adenocarcinoma (LUAD) and liver hepatocellular carcinoma (LIHC), a downregulation of ADM expression was observed in stage IV, potentially due to the limited sample size in the advanced stages of these cancer types.

### 3.3 Pan-cancer mutational landscape of ADM

**Figure 3:**
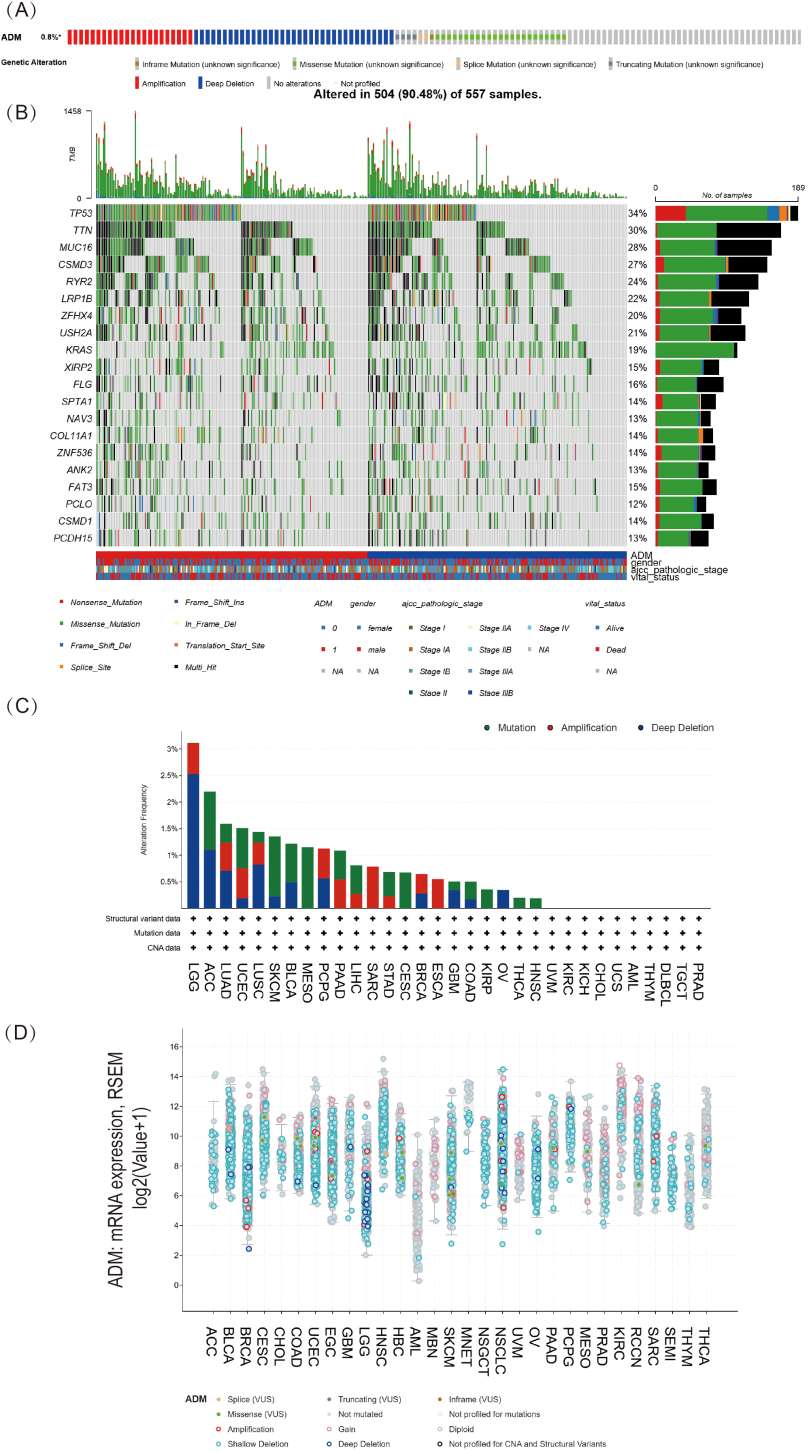
Pan-cancer mutational landscape of ADM. **(A)** The frequency of genetic alterations in ADM across a range of cancer types, representing 0.8% of samples(altered/profiled = 0.8% of 10,967 samples). **(B)** The mutation spectrum grouped by ADM in LUAD with the top 20 most frequently mutated genes presented. **(C)** Stacked bar plot of ADM mutation frequency across various cancer types, with LGG, ACC, and LUAD exhibiting the highest mutation rates. **(D)** Analysis of the correlation between ADM putative copy-number alteration (CNA) and ADM expression in pan-cancer tissues.

The research evaluated the genetic alterations of ADM in multiple cancer types through the cBioPortal platform. As shown in **Figure 3A**, genetic alterations in ADM were observed in 0.8% of 10,967 samples from 10,953 patients with various cancer types. The most common alterations were Amplification and Deep deletion. **Figure 3C** presented a stacked bar chart of ADM mutation frequency across multiple cancers, ordered from highest to lowest mutation rates. LGG, ACC, and LUAD exhibited the highest mutation frequency. In LUAD, the most frequent types of genetic alterations were also Amplification and Deep deletion. Based on these results, as shown in **Figure 3D**, the correlation between ADM’s putative copy-number alteration (CNA) and ADM mRNA expression was investigated. **Figure 3B** displayed the mutation spectrum for high/low ADM expression cohorts of 557 LUAD samples, while the top 20 mutated genes included TP53, TTN, MUC16, CSMD3, RYR2, LRP1B, ZFHX4, USH2A, KRAS, XIRP2, FLG, SPTA1, NAV3, COL11A1, ZNF536, ANK2, FAT3, PCLO, CSMD1, and PCDH15. The mutation analysis of these top 20 genes in relation to high and low ADM expression was shown in **Figure 3B**.

### 3.4 Correlations of ADM expression with CNV and DNA methylation

**Figure 4:**
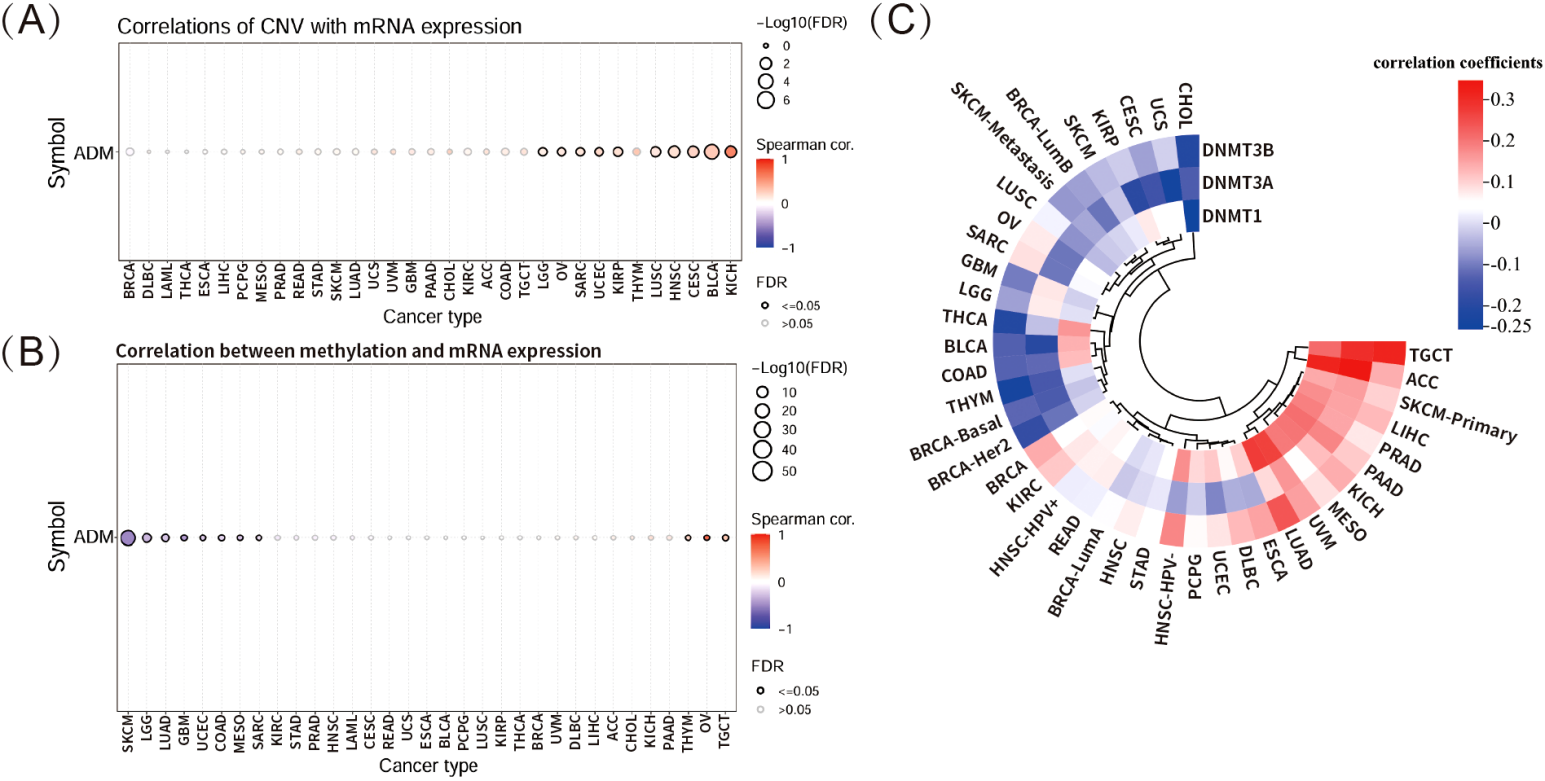
Correlations of ADM expression with CNV and DNA methylation. **(A)** Correlation between CNV and ADM mRNA expression in multiple cancer types via GSCA database. **(B)** Correlation between DNA methylation and ADM mRNA expression in various cancer types via GSCA database. **(C)** Heatmap showing the correlations between ADM expression and three methyltransferases, including DNMT1, DNMT3A, and DNMT3B, across different cancer types.

This study further explored the relationship between copy number variation (CNV) and ADM mRNA expression using the GSCA database (**Figure 4A**). The analysis revealed significant positive correlation between ADM expression and CNV in KICH, BLCA, CESC, HNSC, and LUSC, although the correlation was not strong. In contrast, no significant correlation was observed between CNV and ADM expression in the majority of other cancer types, suggesting that CNV might not be the primary factor driving abnormal ADM expression. Multiple other mechanisms likely contributed to the aberrant expression of ADM in different cancer types.

DNA methylation is a crucial epigenetic process that modulates gene transcription and expression. This study found significantly negative correlation between DNA methylation and ADM mRNA expression in several cancer types, particularly in SKCM (**Figure 4B**). However, in most cancer types, ADM expression did not exhibit significant correlation with corresponding DNA methylation. Furthermore, the correlation heatmap depicting the relationship between ADM expression and three methyltransferase genes (DNMT1, DNMT3A, DNMT3B) in multiple cancer types revealed weak correlations, with small correlation coefficients, indicating that ADM expression was not strongly linked to the activity of these methyltransferase genes at pan-cancer level (**Figure 4C**).

### 3.5 Pan-cancer analysis revealed prognostic significance of ADM expression

**Figure 5:**
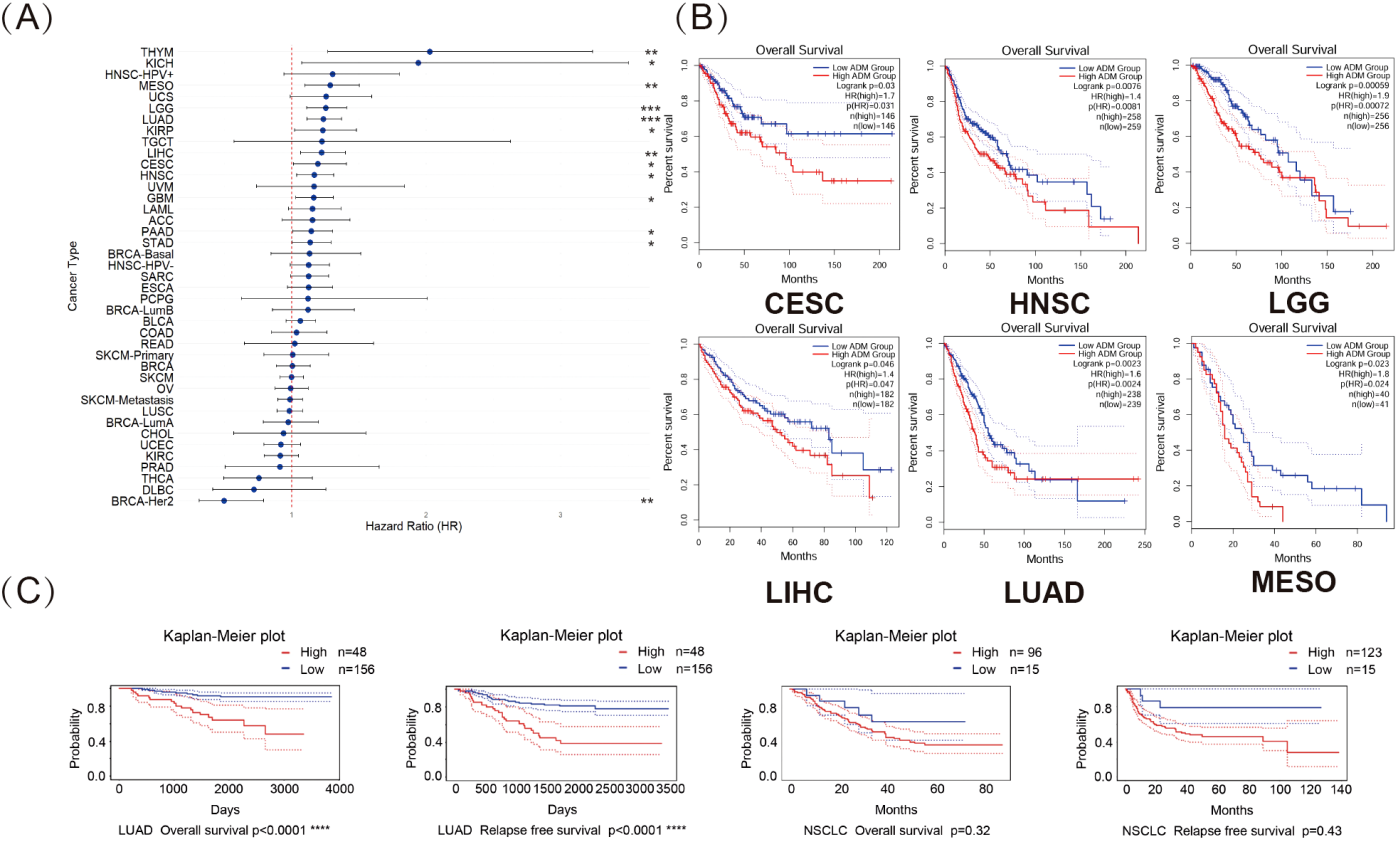
Pan-cancer analysis revealed prognostic significance of ADM expression. **(A)** Forest plot illustrating the impact of high ADM expression on overall survival (OS) across 41 cancer types, with significant associations in LGG, LUAD, MESO, THYM, LIHC, HNSC, GBM, KICH, KIRP, CESC, PAAD, and STAD. (*p<0.05, **p<0.01, ***p<0.001) **(B)** Kaplan-Meier plots for OS, showing the significant prognostic value of elevating ADM expression in CESC, HNSC, LGG, LIHC, LUAD, and MESO. **(C)** Survival analysis from the PrognoScan database, including GSE31210, GSE3141 and GSE8894 datasets, confirming the negative prognostic impact of high ADM expression on overall survival and relapse free survival in LUAD.

To further investigate the impact of ADM expression on the prognosis of patients, this study utilized RNA-seq and clinical data from the TCGA database. Univariate Cox regression analysis was performed to examine the relationship between ADM expression and overall survival (OS) across 41 cancer types. As shown in **Figure 5A**, a forest plot illustrated the risk associated with high ADM expression in various cancers. High ADM expression was significantly linked to poor prognosis in patients with LGG, LUAD, MESO, THYM, LIHC, HNSC, GBM, KICH, KIRP, CESC, PAAD, and STAD, with shorter OS observed in these patients. Notably, the correlation between ADM expression and prognosis was most significant in LGG and LUAD (p<0.0001). In contrast, high ADM expression was associated with better prognosis only in BRCA-HER2. This finding was consistent with the downregulation of ADM in BRCA relative to adjacent normal tissues observed in the previous analysis, suggesting a potential unique protective role of ADM in BRCA. To further evaluate the prognostic potential of ADM expression, survival analysis using GEPIA was conducted for each cancer type, and survival curves were generated. The results in **Figure 5B** revealed that upregulated ADM expression significantly predicted worse prognosis in cancers such as CESC, HNSC, LGG, LIHC, LUAD, and MESO. To validate the predictive significance of elevating ADM expression in LUAD, this research accessed the PrognoScan website and analyzed datasets, GSE31210, GSE3141 and GSE8894, from the GEO database (**Figure 5C**). Kaplan-Meier plots indicated that high ADM expression significantly shortened overall survival (OS) and relapse free survival (RFS) in LUAD patients (p<0.0001), further indicating the role of ADM’s upregulation in the prognosis of LUAD. Meanwhile, upregulated ADM expression was associated with both overall survival and relapse free survival in NSCLC, however, these associations were not statistically significant.

### 3.6 Correlations between ADM expression and immune response markers

**Figure 6:**
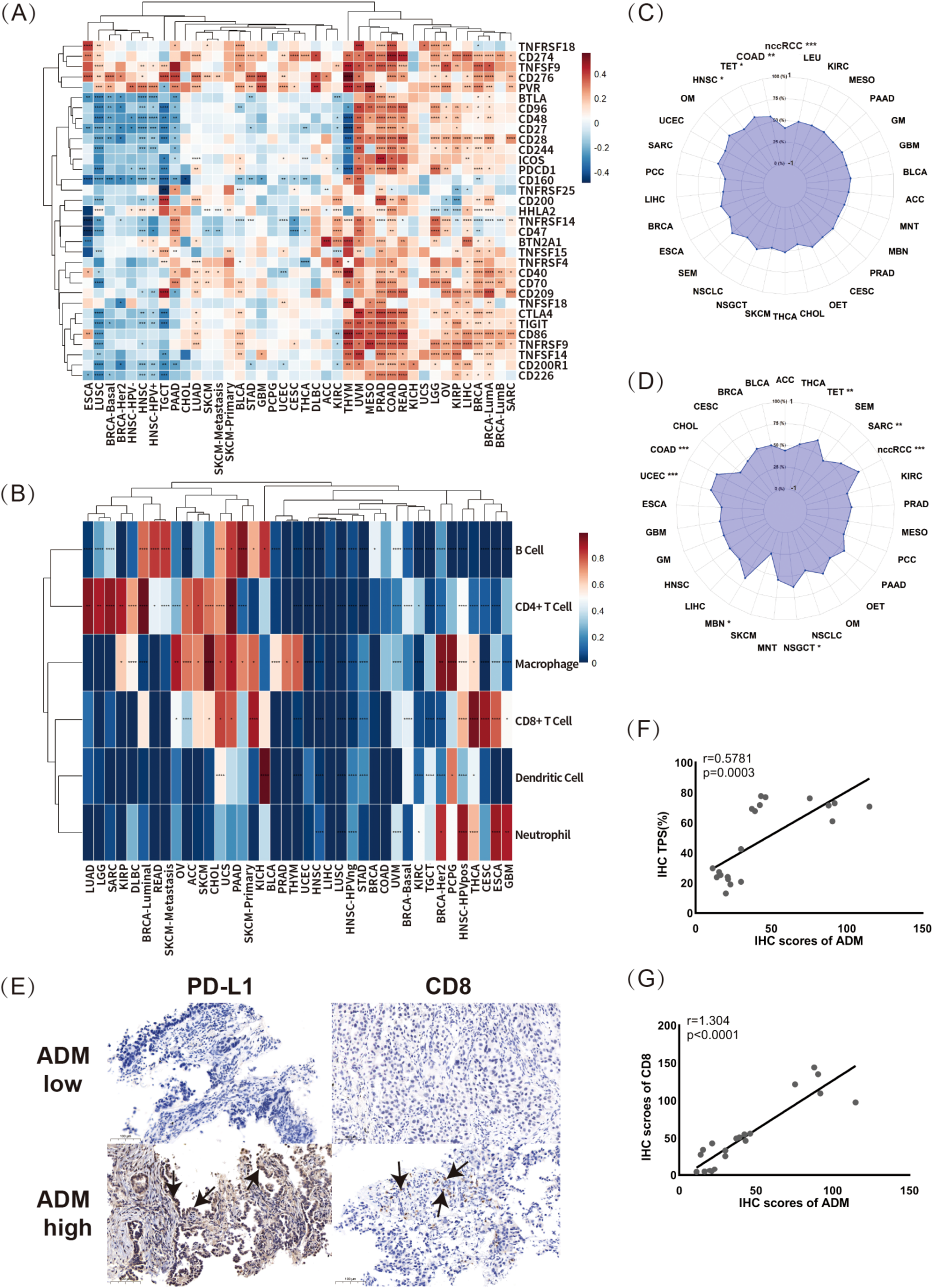
Correlations between ADM expression and immune response markers. **(A)** The correlations between ADM expression and 33 immune-related genes across multiple cancer types (*p<0.05, **p<0.01, ***p<0.001, ****p<0.0001). **(B)** The relationship between ADM expression and immune cell infiltration across various cancer types for B cells, CD4^+^ T cells, CD8^+^ T cells, neutrophils, macrophages, and dendritic cells (*p<0.05, **p<0.01, ***p<0.001, ****p<0.0001). **(C, D)** The correlations of ADM expression with tumor mutational burden (TMB) and microsatellite instability (MSI) in various cancer types. **(E)** Representative IHC staining images showing PD-L1 (CD274) and CD8 expression in LUAD samples with low and high ADM expression. Scale bars, 100 µm. The PD-L1 and CD8 expression regions were marked with arrows. **(F)** Scatter plot and linear fitting of ADM expression and tumor proportion score (TPS) in LUAD tissues. **(G)** Scatter plot and linear fitting of ADM expression and CD8^+^ T cell infiltration in LUAD tissues.

This study initially explored the correlations between ADM expressions and 33 well-known immune-related genes using RNA-seq data from TCGA across various cancers. As shown in the heatmap in **Figure 6A**, the clustering of cancer types based on the correlations between ADM and these 33 immune response-related genes revealed three distinct groups. In cancers such as THYM, UVM, MESO, PRAD, COAD, READ, KICH, UCS, LGG, OV, KIRP, LIHC, BRCA, BRCA-LumA, BRCA-LumB, and SARC, ADM exhibited strong positive correlations with almost all immune-related genes, including BTLA, BTN2A1, CD160, CD200, CD200R1, CD209, CD226, CD244, CD276, CD28, CD40, CD48, CD86, CTLA4, PVR, TIGIT, TNFRSF18, TNFRSF9, TNFSF14, TNFSF18, CD27, CD274, CD70, CD96, ICOS, TNFRSF25, TNFRSF4, TNFSF9, TNFSF15, HHLA2, TNFRSF14, CD47, and PDCD1. Notably, several of these genes, such as PDCD1, TIGIT, CTLA4, and CD276, are known to be associated with T-cell exhaustion, indicating ADM’s potential involvement in immune modulation and T-cell dysfunction. In contrast, in ESCA, LUSC, BRCA-Basal, BRCA-HER2, HNSC-HPV-, HNSC, and HNSC-HPV+, ADM expression presented strong negative correlations with most of the immune-related genes. Notably, in other cancers, including LUAD, there was no significant correlation between ADM and the majority of immune-related genes. However, it was noteworthy that among these immune-related genes, CD274, CD276, TNFRSF18, TNFSF9, and PVR displayed strong correlations with ADM expression across almost all cancer types. Among these five genes, CD274, CD276, and PVR were all validated in various previous researches as suppressive immune modulators, playing significant roles in T cell exhaustion. CD274 (PD-L1), a key immune checkpoint and classic target for immunotherapy, bound to PD-1 on T-cells, leading to their exhaustion in multiple cancer types. Similarly, CD276 (B7-H3) inhibited T-cell activation and contributed to T-cell exhaustion, helping tumors escape immune surveillance. Additionally, PVR (CD155) suppressed T-cell function by interacting with the DNAM-1 receptor, further promoting T-cell exhaustion and immune suppression in the tumor microenvironment. These findings indicated that ADM was tightly linked to immune-related genes, suggesting that it might play an important role in the development of suppressive immune microenvironment, especially in T cell exhaustion. Moreover, a remarkable heterogeneity in the relationship between ADM and immune-related genes was observed across different cancer types, but an intriguing consistency was found in several widely used immune checkpoint genes, CD274, CD276, TNFRSF18, TNFSF9, and PVR, warranting further validation and exploration in basic research.

Building upon this, this study further investigated the relationship between ADM expression and immune cell infiltration using pan-cancer data from the Timer database. The correlations between ADM and six types of tumor-infiltrating immune cells (B cells, CD4^+^ T cells, CD8^+^ T cells, neutrophils, macrophages, and dendritic cells) were visualized in the heatmap as shown in **Figure 6B**. As seen in the figure, multiple cancer types could be largely clustered into two groups based on the correlation between ADM and immune cell infiltration. In cancer types such as LUAD, LGG, SARC, KIRP, MESO, DLBC, BRCA-Luminal, READ, SKCM-Metastasis, OV, ACC, SKCM, CHOL, UCS, PAAD, and SKCM-Primary, ADM illustrated strong positive correlations with B cells, CD4^+^ T cells, and macrophages. In contrast, in GBM, ESCA, CESC, THCA, and HNSC-HPV+, ADM exhibited stronger correlations with CD8^+^ T cells and neutrophils. Additionally, the positive correlations between ADM and CD4^+^ T cells and macrophages were observed across the majority of cancer types, further supporting the potential role of ADM in immune regulation. Moreover, **Figure 6C** and 6D presented the correlations between ADM expression and two important biomarkers for predicting immunotherapy efficacy: tumor mutational burden (TMB) and microsatellite instability (MSI). As shown in the figures, ADM expression was positively correlated with TMB in COAD, TET, and HNSC, while it showed a negative correlation with TMB in nccRCC, although all these correlations were relatively weak (**Figure 6C**). At the same time, ADM expression presented a positive correlation with MSI in nccRCC, COAD, UCEC, TET, SARC, NSGCT, and MEN, while no statistically significant correlation was found in MSI for other cancer types (**Figure 6D**).

Furthermore, to validate these findings, IHC was conducted to explore the relationship between ADM expression and PD-L1 and CD8^+^ T cell infiltration in paired patient samples from before and after EGFR-TKI resistance (**Figures 6E-G**). Scatter plots and linear analysis demonstrated that ADM expression was positively correlated with both the tumor proportion score (TPS) (r=0.58, p=0.0003) and CD8^+^ T cell infiltration (r=1.304, p<0.0001) (**Figures 6F, G**). These results not only supported the idea that ADM expression might contribute to immune modulation in LUAD, but also suggested a potential link between ADM and T-cell exhaustion, given the strong associations with immune checkpoints such as PD-L1 and immune cell infiltration. Specifically, ADM might be involved in shaping an immune suppressive microenvironment, potentially promoting T-cell dysfunction and exhaustion, particularly in the context of EGFR-TKI resistance.

### 3.7 Pan-cancer analysis of correlation between ADM and drug sensitivity

**Figure 7:**
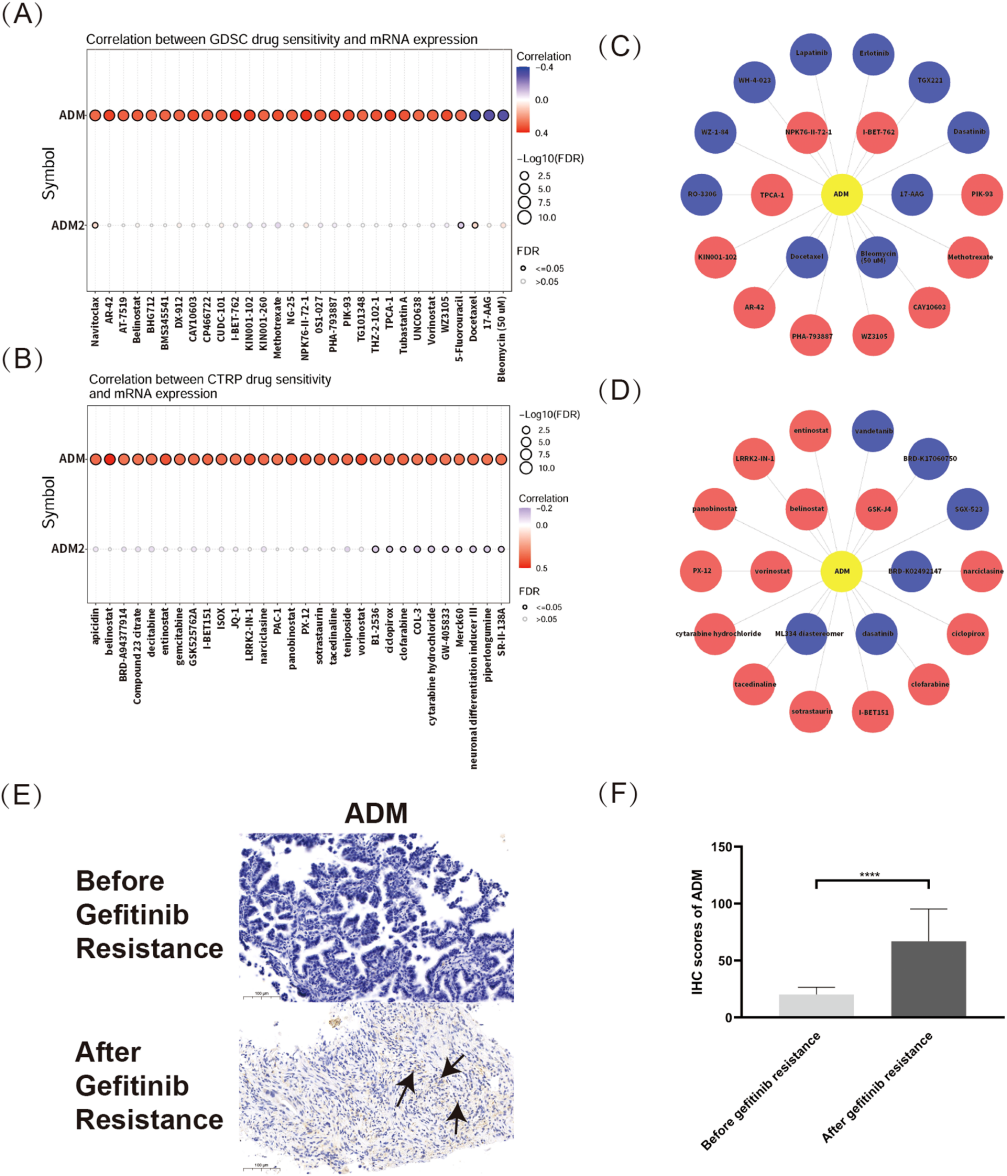
Pan-cancer analysis of correlation between ADM and drug sensitivity. **(A)** Correlation between ADM/ADM2 expressions and drug sensitivity from the GDSC database. **(B)** Relationship between ADM/ADM2 expressions and drug sensitivity from CTRP database. **(C)** Network analysis visualizing the associations between ADM expression and sensitivity to various compounds in the GDSC database. **(D)** Network representation of ADM-associated drug sensitivity based on data from CTRP database. **(E)** Representative IHC staining images of ADM in LUAD tissues before and after EGFR-TKI resistance. Scale bars, 100 µm. The ADM expression regions were marked with arrows. **(F)** Semi-quantitative bar chart of ADM expression levels from the IHC images.

Based on the pan-cancer analysis of drug sensitivity datasets, ADM and ADM2 expression levels were significantly correlated with sensitivity of various medications in both the GDSC and CTRP databases, with ADM showing a broader and more significant correlation. As shown in **Figure 7A**, the top three medications positively correlated with ADM expression in the GDSC database were I-BET-762, NPK76-II-72-1, and TPCA-1. I-BET-762 (molibresib) had been widely studied as a BRD4 Inhibitor for its therapeutic potential in various malignancies (34, 35), while TPCA-1 had been extensively used in infectious diseases for its anti-inflammatory effect. Besides, NPK76-II-72-1, as an experimental compound, had also been investigated for its role in glioblastoma and hepatocellular carcinoma (36, 37). Meanwhile, the top three negatively correlated medications were 17-AAG, bleomycin (50 uM), and docetaxel, indicating ADM’s role in the development of chemotherapy resistance. Given the potential for online databases to overlook medications with moderate correlations with ADM, further analysis using the GDSC database was conducted, presenting the relationship between ADM expression and drug sensitivity in a network format. **Figure 7C** highlighted that, in addition to the aforementioned medications, ADM expression was also negatively correlated with RO-3306, WZ-1-84, WH-4-023, TGX221, and several tyrosine kinase inhibitors (TKIs), such as lapatinib, erlotinib, and dasatinib, which were crucial in anti-tumor therapy.

Besides, the CTRP database results, shown in **Figure 7B**, revealed that ADM and ADM2 expression levels were correlated with a variety of antitumor medications. The top three medications positively correlated with ADM expression were GSK-J4, belinostat, and vorinostat, while negatively correlated medications included BRD-K02492147, dasatinib, and ML334 diastereomer. Additionally, **Figure 7D** illustrated ADM’s relationship with sensitivity to various antitumor medications from the CTRP database using a network graph, providing an overview of ADM’s influence on drug sensitivity. These findings also indicated that ADM expression was closely related to the sensitivity of a wide range of antitumor medications, particularly histone deacetylase inhibitors (e.g. vorinostat and entinostat) and tyrosine kinase inhibitors (TKIs) (e.g. dasatinib and vandetanib). Interestingly, ADM’s negative correlations with various TKIs, for example, erlotinib, lapatinib, dasatinib and vandetanib, were validated in both two databases, demonstrating its pivotal role in the development of TKI resistance in multiple cancer types. All these results suggested that ADM could be an important factor in predicting drug sensitivity and guiding personalized cancer therapy.

To specifically address the concern regarding tumor-specific analysis, IHC staining was conducted based on patients’ samples to explore the relationship between ADM expression and EGFR-TKI resistance in LUAD, a cancer type that is highly relevant to our study. Our results revealed a significant upregulation of ADM expression in tumor tissues after resistance to EGFR-TKI treatment (**Figures 7E-F**). This finding directly validated the role of ADM in drug resistance in LUAD, particularly in the context of TKI resistance.

### 3.8 ADM-associated pathways and functions in LUAD

**Figure 8:**
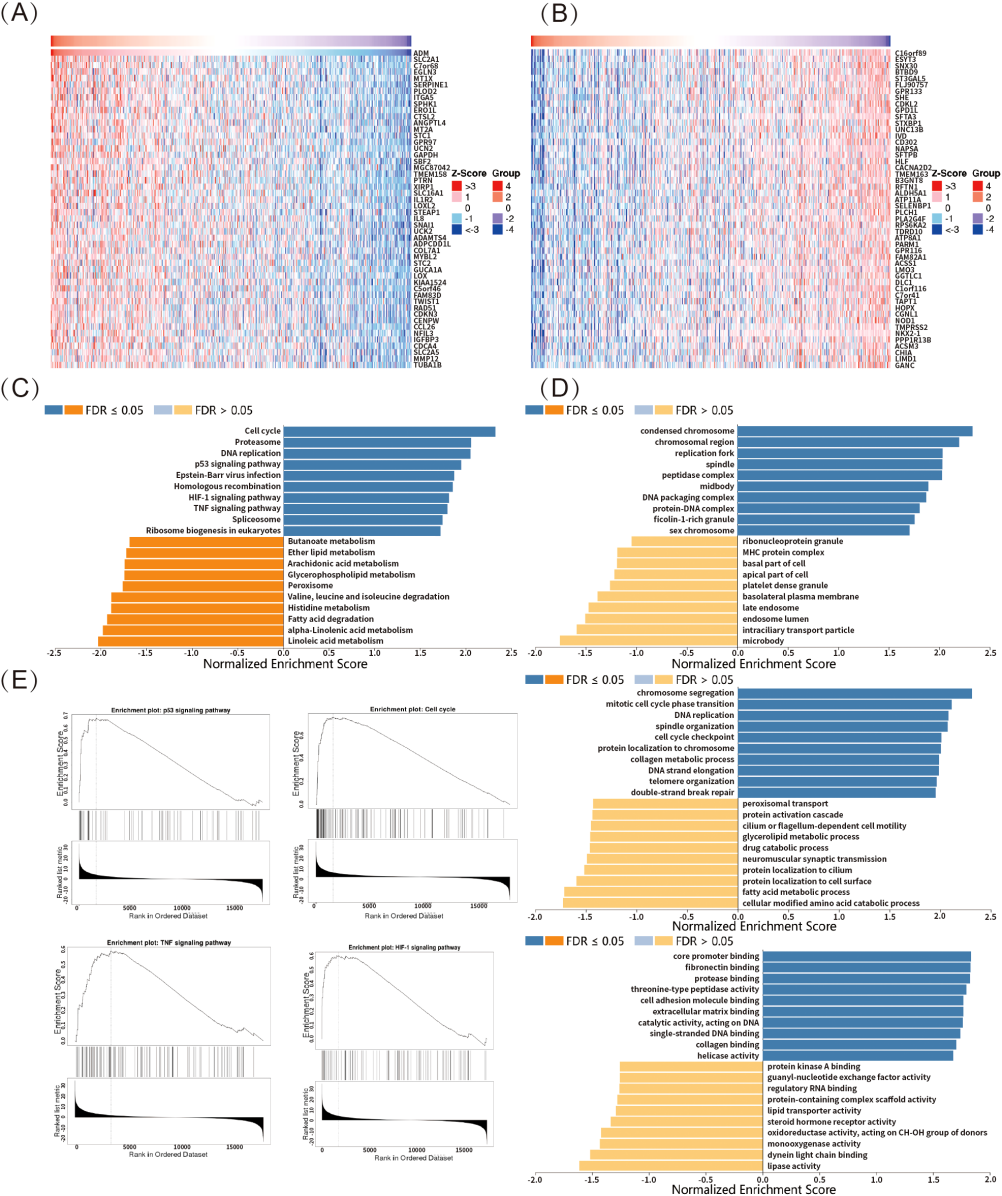
ADM-associated pathways and functions in LUAD. **(A, B)** Heatmaps showing the top 50 significant genes positively and negatively correlated with ADM expression in LUAD. **(C)** KEGG pathway enrichment analysis revealed that ADM-associated genes were enriched in pathways such as p53 signaling, HIF-1 signaling, TNF signaling, cell cycle, and DNA replication. **(D)** GO enrichment of ADM-associated genes highlighted processes relating to DNA repair, chromatin regulation, and cell cycle modulation. **(E)** Representative ADM-associated genes enrichment plots: p53 signaling, cell cycle, HIF-1 signaling and TNF signaling.

In this study, LUAD was used as a representative example to explore ADM-related genes through the LinkedOmics online platform. The top 50 significant genes positively or negatively correlated with ADM expression were shown in **Figure 8A** and 8B, respectively. GSEA-based gene enrichment analysis of LUAD RNA-seq data was revealed in **Figures 8C-E**. GO enrichment results, categorized by biological process, cellular component, and molecular function, were primarily associated with DNA and chromatin repair, epigenetic regulation, as well as the cell cycle modulation (**Figure 8D**). KEGG pathway enrichment analysis demonstrated that, in addition to cell cycle and DNA replication, the main enriched pathways included the p53 signaling pathway, HIF-1 signaling pathway, and TNF signaling pathway (**Figures 8C, E**). These pathways were known to play critical roles in oncogenesis, tumor progression, resistance, and immune modulation. Together with the previous findings on ADM’s relationship with immune response markers and drug sensitivity, these results further highlighted ADM’s potential as a key target for research into tumor immunity and resistance.

## 4 Discussion

Adrenomedullin (ADM) is a multifunctional peptide that plays a pivotal role in various pathological processes, particularly in cancer progression. It was overexpressed in a wide range of cancer types, where it promoted oncogenesis, metastasis, angiogenesis, and immune evasion (38–40). Despite the complex role of ADM across different cancers, a comprehensive pan-cancer analysis examining its impact on prognosis, immune microenvironment, and resistance to anti-tumor therapies, specifically chemotherapy and TKI therapies, had been lacking. This study aimed to address these gaps by investigating ADM across various cancer types, providing insights into its potential as a prognostic biomarker and a therapeutic target for precise medicine. The findings of this research demonstrated that ADM served as a negative prognostic biomarker in several cancer types, with a particularly significant impact in lung adenocarcinoma (LUAD). Moreover, ADM was found to contribute to the development of a suppressive immune microenvironment and to the development of resistance to TKI treatment, a finding that was further validated in LUAD.

In this study, a comprehensive pan-cancer analysis of ADM expression and its correlation with clinical outcomes was performed. ADM was found to be significantly upregulated in several cancer types, including CESC, ESCA, GBM, HNSC, KICH, KIRC, LUSC, PCPG, THCA, and UCEC. Our study further presented that ADM expression was correlated with advanced pathological stages in cancers such as THCA, PAAD, LUAD, LIHC, KIRP, and HNSC, suggesting its role in disease progression. These findings aligned with previous studies demonstrating the overexpression of ADM in various malignancies and its role in promoting tumor progression through angiogenesis (28), immune modulation (40), and enhancing tumor cell survival (41). As an autocrine and paracrine growth factor, ADM was regulated by hypoxia inducible factor 1 (HIF-1) in hypoxic condition, a hallmark of solid tumors (13, 28). Several studies clarified that this regulation promoted neovascularization and contributed to aggressive tumor phenotypes in ovarian and pancreatic cancers (38, 39). Moreover, other researches elucidated that ADM inhibited apoptosis, thereby promoting tumor cell survival (42, 43). However, discrepancies with prior research were observed in certain cancer types. For example, ADM was identified to be downregulated in BRCA, contrasting with studies linking ADM to increased tumor aggressiveness in various breast cancer subtypes (44). These discrepancies might be attributed to subtype-specific variations in ADM’s regulatory mechanisms or its interactions with distinct immune and stromal components within the tumor microenvironment (TME). This variability highlighted the importance of considering cancer type and context when evaluating the function of ADM.

Notably, this study explored the relationship between ADM expression and immune-related genes across a range of cancer types, identifying distinct correlation patterns. ADM was found to exhibit a strong and consistent positive correlation with several immune checkpoint genes, including CD274 (PD-L1), CD276, TNFRSF18, TNFSF9, and PVR in the pan-cancer analysis, which contributed to the development of a suppressive immune microenvironment. These findings were consistent with previous studies on autoimmune diseases, which demonstrated that ADM could modulate the immune system by promoting immune tolerance and suppressing T cell activity, as observed in rheumatoid arthritis and sepsis-related immune responses (45, 46). Although some studies had explored the relationship between ADM and immune cells in other inflammatory diseases, research on the association between ADM and immune microenvironment, particularly immune checkpoint-related genes, from the pan-cancer aspect, remained limited. Therefore, this study presented, for the first time, a comprehensive analysis of the relationship between ADM and 33 common immune checkpoint-related genes. Based on these correlations, cancer type clustering was performed, offering a clear depiction of the heterogeneity of ADM’s correlation with immune checkpoint genes across various cancer types. These findings lay a foundation for further basic and clinical research in this field.

The relationship between ADM expression and tumor-infiltrating immune cells was also analyzed. This research revealed strong positive correlations of ADM with CD4^+^ T cells, macrophages, and B cells in cancers such as LUAD and MESO, supporting ADM’s role in recruiting and activating immune cells. These results were also consistent with findings in inflammatory and autoimmune diseases, where ADM influenced macrophage polarization and regulatory T cell activity (47, 48). Notably, in cancers like GBM and HNSC, ADM exhibited stronger correlations with CD8^+^ T cells and neutrophils, suggesting varied immunological roles depending on the cancer type. In addition, our study further demonstrated that ADM expression was positively associated with tumor mutational burden (TMB) and microsatellite instability (MSI) in several cancers, including COAD and TET, suggesting ADM’s potential as a biomarker for predicting the efficacy of immunotherapy. Similar associations had been reported in other studies, where ADM’s interaction with immune-related biomarkers influenced outcomes in various cancer types. For example, the ADM-RAMP signaling axis had been identified as a critical mediator of angiogenesis and metastasis, with distinct roles of RAMP2 and RAMP3 in regulating TME (49–51).

Moreover, a comprehensive analysis was done on the relationship between ADM expression and drug sensitivity using data from the GDSC and CTRP databases. ADM expression demonstrated significant correlations with the sensitivity of various anti-tumor medications. Notably, ADM expression was negatively correlated with several critical medications used in chemotherapy, for example, bleomycin and docetaxel, and several tyrosine kinase inhibitors (TKIs), such as lapatinib, erlotinib, and dasatinib, suggesting ADM’s potential role in mediating resistance to chemotherapy and targeted therapy. Our findings validated and extended the results of multiple previous researches highlighting ADM’s involvement in resistance. For instance, studies in ovarian cancer had shown that ADM promoted platinum-based chemotherapy resistance by upregulating glycolysis via PKM2, consistent with our observation of ADM’s negative correlation with certain chemotherapeutic agents (35, 52). Similarly, the ADM-HIF-1α axis had been implicated in enhancing chemoresistance in breast cancer (28). However, discrepancies were also noted when evaluating ADM’s role across different cancer types. For example, while ADM expression was associated with resistance in ovarian and breast cancers, it acted as a potential biomarker for enhancing sensitivity to glycolysis-targeting agents in gliomas (14). Such differences might be attributed to tumor-specific metabolic dependencies and distinct tumor microenvironment characteristics. Besides, no studies had yet explored the relationship between ADM and resistance to EGFR-TKIs or other TKIs in tumors. Our study elucidated that ADM was intricately linked to drug sensitivity, particularly with chemotherapy and TKI therapy, suggesting its role as a key regulator of therapy response. These findings underscored ADM’s potential as a predictive biomarker and therapeutic target for overcoming drug resistance, while the observed heterogeneity called for further research to delineate ADM’s cancer-specific functions and interactions within the therapeutic landscape.

Although this study provided meaningful insights, several limitations should be acknowledged. Firstly, the data primarily relied on bioinformatics tools and publicly available databases, which underscored the need for further experimental validation to confirm these findings, especially the identified pathways related to ADM. Secondly, while ADM’s role in immune regulation and therapy response was evident, the underlying molecular mechanisms remained to be fully explored. Thirdly, single-cell transcriptomic data should be used to analyze the relationship between ADM and immune microenvironment. Due to the limited scope of current pan-cancer datasets, the immune-related analyses largely depended on the TIMER algorithm. Additionally, although the relationship between ADM and TKI resistance at the cellular level in LUAD had been explored, further experiments were required to investigate the detailed mechanisms behind the correlation between ADM expression and IC50 of TKIs in LUAD.

## 5 Conclusion

This pan-cancer analysis revealed ADM’s pivotal role in progression, immune modulation, and therapeutic response, especially in LUAD. ADM held promise as a prognostic biomarker and a potential therapeutic target. Future research should focus on experimental validation and elucidation of ADM-mediated pathways, which might provide novel insights into cancer biology and improve clinical outcomes.

## Data Availability

The original contributions presented in the study are included in the article/supplementary material. Further inquiries can be directed to the corresponding author.
